# Disability and Non-Motor Symptoms in Multiple Sclerosis: Exploring Associations and Predictive Factors

**DOI:** 10.3390/brainsci15101122

**Published:** 2025-10-18

**Authors:** Ana Jerković, Ivona Stipica Safić, Sanda Pavelin, Nikolina Pleić, Klaudia Duka Glavor, Igor Vujović, Joško Šoda, Jasna Duranović, Maja Rogić Vidaković

**Affiliations:** 1 Laboratory for Human and Experimental Neurophysiology (LAHEN), Department of Neuroscience, School of Medicine, University of Split, 21000 Split, Croatia; anasuto@gmail.com (A.J.); jasna.duranovic@mefst.hr (J.D.); 2 Department of Family Medicine, School of Medicine, University of Split, 21000 Split, Croatia; istipica@gmail.com; 3 Department of Neurology, University Hospital of Split, 21000 Split, Croatia; spavelin@gmail.com; 4 Department of Biology and Human Genetics, School of Medicine, University of Split, 21000 Split, Croatia; npleic@mefst.hr; 5 Department of Health Studies, University of Zadar, 23000 Zadar, Croatia; klaudia.dukaglavor@gmail.com; 6 Department of Neurology, General Hospital Zadar, 23000 Zadar, Croatia; 7 Department for Marine Electrical Engineering and Information Technologies, Faculty of Maritime Studies, University of Split, 21000 Split, Croatia; ivujovic@pfst.hr (I.V.); jsoda@pfst.hr (J.Š.)

**Keywords:** multiple sclerosis, sleep, fatigue, depression, anxiety, disability evaluation

## Abstract

Background/Objectives: The relationship between multiple sclerosis (MS) disability and co-occurring non-motor symptomatology is not well understood. This study examined the association between disability status and non-motor symptoms—sleep quality, depression, anxiety, and fatigue—in people with multiple sclerosis (MS), as well as the contribution of sleep quality to the prediction of fatigue, depression, and anxiety in MS. Methods: A cross-sectional study included 469 MS and 369 control subjects. Disability status of MS subjects was assessed with the Expanded Disability Status Scale (EDSS), while fatigue, depression, anxiety, and sleep quality were evaluated with the Fatigue Severity Scale (FSS), the Hospital Anxiety and Depression Scale (HADS), and the Pittsburgh Sleep Quality Index (PSQI), respectively. Statistical analyses encompassed group comparisons, Pearson correlations, and hierarchical regression models adjusted for age, sex, and EDSS. Results: The results show that MS subjects exhibited higher FSS, HADS-D, and PSQI scores than controls, with intercorrelations and only weak associations with EDSS severity (r = 0.15–0.29). Moreover, PSQI global and HADS-D scores increased with higher EDSS severity, while FSS scores peaked in the moderate EDSS range (4.5–6.5). Global PSQI score independently predicted FSS, HADS-D, and HADS-A. Daytime dysfunction, sleep disturbances, and sleep medication use significantly predicted FSS, HADS-D, and HADS-A scores. Conclusions: Study findings highlight the role of sleep quality in exacerbating depression, anxiety, and fatigue in MS.

## 1. Introduction

Multiple sclerosis (MS) is a chronic, disabling neurological autoimmune disorder of the central nervous system (CNS) characterized by demyelination, neuroinflammation, and neurodegeneration [1,2,3,4,5,6], and it affects 1.89 million people, with the global prevalence of 23.9 cases per 100,000 population (Khan & Hashim, 2025). MS organizations (National Multiple Sclerosis Society) estimate that 2.8 million people suffer from MS, with an estimated number of 500,000–700,000 MS in Europe [7].

Extensive pathology in the gray matter of the cerebral cortex and other CNS regions is thought to be responsible for various complex neurological indices associated with disease progression in motor and sensory systems [2,4], lower urinary tract symptoms and related comorbidities [8], chronic pain [9], disruption in cognitive and psychological functioning, and increased level of fatigue [2,4,10,11,12,13,14]. Depression, anxiety, and stress are frequent psychological symptoms of MS (prevalence of 25–65%, 20–54%, and 44.8%, respectively) [15,16], often associated with fatigue (prevalence from 36.5 to 78.0%) [17], cognitive impairment (prevalence of 45–70%) [15], and sleep disorders (up to 67% more common than in the general community) [18]. Emerging evidence positions sleep disturbances as key mediators and moderators, exacerbating psychological distress and fatigue independently of disability progression [19]. These disturbances include poor sleep initiation, reduced duration, and efficiency [19], alongside polysomnographic abnormalities such as diminished stage N2 sleep [20]. Critically, sleep quality independently clusters with MS symptoms and predicts quality of life [18], while depression, anxiety, and stress explain 37% of sleep disorder variance and 35% of fatigue risk [21]. Despite their interconnectedness, co-occurrence symptoms are overlooked by the Expanded Disability Status Scale (EDSS) [22].

The EDSS is based on a clinical neurological examination, including seven functional systems (FS: visual, brainstem, pyramidal, cerebellar, sensory, bowel/bladder, and cerebral) and assessment of walking range (ambulation) [23,24]. Regarding the cerebral FS score, depression is not considered in FS and EDSS calculations, and fatigue evaluation is often omitted due to the neurologist’s difficulties in objectively assessing fatigue. Despite being the most widely used clinical tool in MS clinical trials, EDSS has limitations, including less sensitivity in assessing symptoms of depression, anxiety, fatigue, cognitive disturbances, and difficulties in upper limb function [25,26].

The relationship between EDSS scores and co-occurring symptoms remains inconsistent across studies. While some research demonstrates a significant association between higher EDSS scores and increased levels of depression, anxiety, sleep problems, and fatigue [19,27,28,29], other studies report no clear correlation [30,31]. Importantly, even when statistically significant, correlations between EDSS scores and co-occurring symptoms in MS are generally low, highlighting the limitations of the EDSS, which somehow prioritizes motor function and ambulation over non-physical symptoms [2,11,26,30]. In clinical practice, patient-reported measures such as depression, anxiety, and fatigue are collected by only 28–32% of European MS registries, whereas EDSS and other disability measures are routinely included [7].

Given the limitations of EDSS and inconsistent findings on the relationship between co-occurring symptoms in MS, the present study aims to address this gap by comparing the relationship of MS disability status with non-motor symptoms (depression, anxiety, fatigue, and sleep quality) in MS subjects. Notably, sleep problems have been shown to independently predict non-motor symptoms, mediating the effects of disability on mood and quality of life [18]. Therefore, the study also examines the contribution of individual components of sleep quality (assessed by the Pittsburgh Sleep Quality Index, PSQI) [32], concerning MS disability status (EDSS), depression and anxiety (evaluated with the Hospital Anxiety and Depression Scale, HADS) [33], and fatigue (evaluated with the Fatigue Severity Scale, FSS) [34].

## 2. Materials and Methods

### 2.1. Study Procedure

A cross-sectional study design using a mixed-methods approach was used; therefore, online surveys were combined with paper-based questionnaires. Paper-based questionnaires were given to MS subjects at regular neurologic visits at the Department of Neurology at the University Hospital Split (*n* = 115) and the Department of Neurology, General Hospital Zadar (*n* = 354). This study included a total of 469 MS subjects, members of the Association of Multiple Sclerosis Societies of Croatia (AMSSC). The control subjects were recruited through online social media communities (*n* = 369).

Participation in the study was voluntary and anonymized, with exclusion criteria encompassing a history of psychiatric disorders or other neurological conditions other than MS. During screening, 5.54% of MS subjects and 1.31% of control subjects were excluded (e.g., psychiatric and neurological disorders). Data collection spanned from 1 October to 30 November 2024.

### 2.2. Demographic Information and Disease-Related Variables

Demographic characteristics (age, sex, and handedness), educational attainment, comorbidities, and medications for comorbid conditions were recorded for all participants. For individuals with MS, disease-specific data were collected, including disease duration, MS subtype, EDSS scores, and current immunomodulatory therapies.

### 2.3. Participants

Demographic and clinical data are presented in Table 1. The mean age of individuals with MS was 42.7 ± 10.8 years (range: 18–78), with females comprising 85% of the cohort. The majority of subjects with MS had completed high school (53.7%), while 31.1% held a graduate degree.

According to the McDonald criteria [35,36], the distribution of MS subtypes was as follows: relapsing-remitting MS (75.3%), primary progressive MS (12.8%), secondary progressive MS (7.8%), and Clinically Isolated Syndrome (CIS) (0.4%). The median EDSS score was 2 [Q1–Q3: 1–4], and the mean disease duration was 8.39 ± 7.74 years. Immunomodulatory therapy was administered to 72.9% of MS subjects, with comorbidities reported in 29% of MS subjects, most commonly endocrine, metabolic, and circulatory system disorders.

The control group consisted of 369 participants with a mean age of 42.3 ± 11.7 years (range: 18–75), of whom 82% were female. Graduate degrees were held by 61.2%, while 23.3% had completed high school. Comorbidities were present in 25.7% of control subjects, predominantly endocrine, metabolic, and circulatory disorders.

### 2.4. Questionnaires

#### 2.4.1. Fatigue Severity Scale (FSS)

The FSS, developed by Krupp et al. [34], is a 9-item questionnaire assessing subjective fatigue perception. Each item is rated on a 7-point Likert scale (1 = strong disagreement to 7 = strong agreement), with total scores calculated as the mean of all items. Fatigue in MS is a multidimensional symptom, encompassing physical, cognitive, and psychosocial aspects. However, most widely used fatigue scales, such as the Fatigue Severity Scale (FSS), primarily measure the severity and impact of physical fatigue, with limited coverage of cognitive or psychosocial fatigue [37,38]. The validated Croatian version demonstrated excellent internal consistency (Cronbach’s α = 0.93) and a unidimensional structure [39]. A cut-off score of ≥4 identifies clinically significant fatigue in MS subjects, with high sensitivity and specificity [40,41,42].

#### 2.4.2. Hospital Anxiety and Depression Scale (HADS)

The HADS, developed by Zigmond and Snaith [33], comprises two seven-item subscales measuring anxiety (HADS-A) and depression (HADS-D). In MS subjects, the HADS has demonstrated robust psychometric properties. Honarmand and Feinstein [43] found that a cut-off score of eight or greater on either subscale provides high sensitivity and specificity for detecting major depression and generalized anxiety disorder, confirming the scale’s utility as a screening tool in this population. These findings have been supported by subsequent studies, which highlight the HADS’ effectiveness for identifying clinically significant psychiatric symptoms in MS [44,45,46].

The Croatian version of the HADS exhibits excellent internal consistency (Cronbach’s α = 0.82–0.83) and supports a two-factor structure, aligning with the original scale’s design [47]. Psychometric analyses further confirm good convergent and incremental validity, with significant correlations observed between HADS subscales and measures of MS impact. For the Croatian MS sample, optimal cut-off scores have been identified as >7 for anxiety and >6 for depression, slightly lower than the original threshold, to maximize diagnostic accuracy in this specific population [47].

#### 2.4.3. The Pittsburgh Sleep Quality Index (PSQI)

The PSQI, developed by Buysse et al. [32], evaluates sleep quality across seven components: sleep quality, sleep latency, sleep duration, sleep efficiency, sleep disturbances, sleep medication use, and daytime dysfunction. Most items use a 4-point scale (subscores 0–3), while four require numeric responses. The global score (0–21) is derived from subscores, with >5 indicating poor sleep quality [48]. Jerković et al. [39] validated PSQI on the MS population, showing excellent reliability (Cronbach’s α = 0.83) and a two-factor structure.

### 2.5. Statistical Analyses

All statistical analyses were performed using R version 4.1.3. [49]. Descriptive statistics were calculated to summarize demographic, clinical, and questionnaire-based variables. Group differences (MS vs. controls) were assessed using independent t-tests (continuous variables) and χ^2^ tests (categorical variables), as well as the Kruskal–Wallis H test for comparisons across more than two groups defined by disability severity. The classification of EDSS scores into mild (0–4), moderate (4.5–6.5), and severe (7–10) is based on the degree of disability and mobility impairment [24]. Average standardized scores (z-values) for the PSQI, FSS, HADS-A, and HADS-D were used to depict the distribution of symptom severity across EDSS categories, serving as the basis for the graphical presentation.

Given the exploratory nature of this study, the analyses were designed to examine potential associations among variables rather than to test specific hypotheses. Pearson correlations examined relationships between FSS, HADS-A, HADS-D, PSQI components, and EDSS in the MS cohort. Hierarchical linear regression models assessed associations of sleep quality with depression, anxiety, and fatigue. Models included blocks with predictors and covariates (age, sex, and EDSS). Results are reported as unstandardized coefficients (β) and adjusted R^2^. Controls were excluded from these models. Causal inference methods were not applied due to the cross-sectional design of the study, which limits the ability to establish cause-and-effect relationships. The level of statistical significance was set at 0.05.

## 3. Results

### 3.1. Group Differences and Symptom Interrelations

The demographic characteristics and disease-related variables of MS and the control group subjects are presented in Table 1. No statistically significant differences were observed in sex (*p* = 0.384) or age distribution (*p* = 0.582) between the examined groups (Table 1). Subjects with MS showed significantly higher FSS (5.01 ± 1.68 vs. 4.02 ± 1.37; *p* < 0.001), HADS-D (7.02 ± 4.31 vs. 5.48 ± 3.51 in controls), and PSQ global scores compared to control subjects (PSQI: 7.75 ± 4.04 vs. 5.97 ± 3.14; *p* < 0.001) (Table 2). HADS-A scores did not differ between groups (*p* = 0.961) (Table 2).

In the MS group, moderate to strong intercorrelations were observed between FSS, HADS-A, HADS-D, and PSQI global (Table 3). EDSS scores were significantly, but weakly associated with FSS (r = 0.266, <0.001), HADS-D (r = 0.294, <0.001), and PSQI global (r = 0.153; *p* < 0.004), while the association of EDSS with HADS-A did not reach statistical significance (*p* = 0.056, *p* > 0.05).

The results show that mean scores for HADS-D (H = 15.07, *p* < 0.001), FSS (H = 13.56, *p* < 0.001), and PSQI (H = 6.35, *p* = 0.002) were higher in MS subjects with more severe EDSS levels, whereas anxiety (HADS-A) showed no significant differences (H = 2.45, *p* = 0.088). Mean (SD) scores for depression (HADS-D) increased with greater EDSS severity: from 6.51 (±4.27) in the mild group, to 8.93 (±4.41) in the moderate group, and 8.73 (±4.92) in the severe group. Similarly, mean (SD) FSS rose from 4.88 (±1.67) in mild cases to 5.74 (±1.41) in moderate and 5.73 (±1.83) in severe cases. The global mean (SD) PSQI scores also showed a progressive increase, rising from 7.55 (±3.21) in the mild group to 8.91 (±3.45) in the moderate group and reaching 10.33 (±4.12) in the severe group.

**Figure 1:**
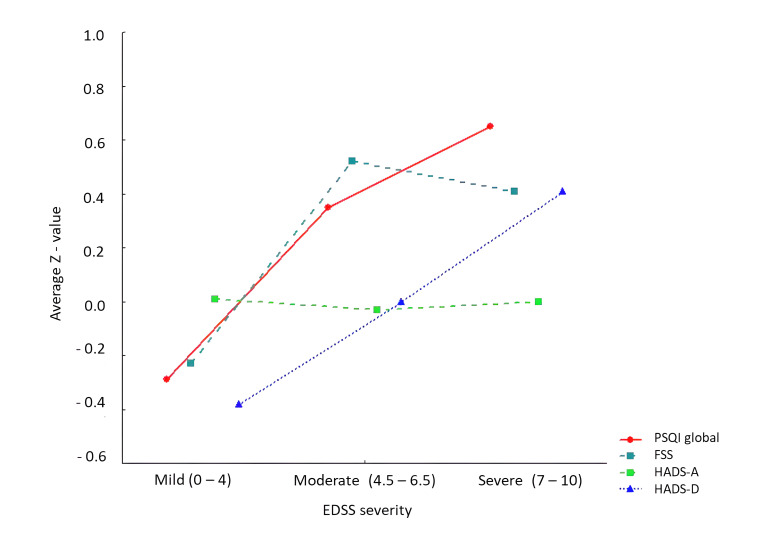
Average standardized scores (Z-values) of FSS, HADS-A, HADS-D, PSQI across EDSS severity stratum. Legend: FSS(Fatigue Severity Scale); HADS-A (Hospital Anxiety and Depression Scale—anxiety subscale); HADS-D (Hospital Anxiety and Depression Scale—depression subscale); PSQI (Pittsburgh Sleep Quality Index); EDSS (Expanded Disability Status Scale).

Figure 1 shows that average standardized PSQI global scores and HADS-D scores increased gradually with higher EDSS severity scores. FSS increased markedly in the moderate EDSS severity stratum and remained elevated at severe EDSS, while HADS-A scores showed minimal variation across EDSS severity levels.

### 3.2. Regression Analysis

Hierarchical linear regression analyses revealed global PSQI scores as a robust and consistent predictor of anxiety symptoms (HADS-A) across all models (Table 4). In the unadjusted model (Model 1), PSQI scores demonstrated a strong positive association with HADS-A (β = 0.528, *p* < 0.001), explaining 22.3% of the variance in HADS-A outcomes. Subsequent adjustment for age and sex (Model 2) yielded negligible attenuation in effect size (β = 0.519, *p* < 0.001), with the model retaining comparable explanatory power (adjusted R^2^ = 0.220).

The fully adjusted model (Model 3), which incorporates EDSS alongside demographic covariates, further confirms the stability of this relationship. PSQI scores remained a significant predictor of HADS-A (β = 0.527, *p* < 0.001), with the final model accounting for 24% of the variance in anxiety symptoms. Age, sex, and EDSS were not statistically significant predictors in the final model.

Further analyses confirmed the global PSQI score as a stable predictor of HADS-D across all models (Table 5). In the unadjusted model (Model 1), the PSQI demonstrated a strong positive association with depression (β = 0.499, *p* < 0.001), accounting for 21.5% of the variance in outcomes (adjusted R^2^ = 0.215). After adjusting for age and sex (Model 2), the strength of the association remained unchanged (β = 0.489, *p* < 0.001), with age emerging as a significant covariate (β = 0.051, *p* = 0.003), indicating that higher age was associated with greater depressive symptom severity.

In the fully adjusted model (Model 3), which included EDSS, the global PSQI score retained its statistical significance as an independent predictor (β = 0.480, *p* < 0.001). Simultaneously, EDSS showed a robust association with HADS-D (β = 0.435, *p* < 0.001). The final model accounted for 24.8% of the variance in HADS-D scores, with PSQI remaining a key factor within the multivariate analysis.

Hierarchical regression analyses identified global PSQI scores as a significant predictor of FSS across all models (Table 6). In the unadjusted model (Model 1), poorer PSQI demonstrated a strong positive association with fatigue (β = 0.166, *p* < 0.001), explaining 15.7% of the variance in FSS outcomes. Controlling for age and sex (Model 2) resulted in a minor reduction in the sleep-fatigue association (β = 0.153, *p* < 0.001), as neither demographic factor demonstrated independent predictive significance in the adjusted model.

The fully adjusted model (Model 3), incorporating EDSS, revealed two key findings: (1) the PSQI global score remained a robust independent predictor of fatigue (β = 0.151, *p* < 0.001), and (2) EDSS emerged as a significant contributor to fatigue severity (β = 0.170, *p* < 0.001). Notably, male sex exhibited an inverse relationship with fatigue in this final model (β = −0.484, *p* = 0.024), suggesting potential sex-specific moderators in fatigue etiology.

Three separate multiple regression models were conducted to examine whether individual PSQI components predicted HADS-A, HADS-D, and FSS while adjusting for age, sex, and EDSS. Across all three outcomes, daytime dysfunction (PSQI7) was the most consistent and robust predictor, significantly associated with higher scores on HADS-A (β = 1.990, *p* < 0.001), HADS-D (β = 2.220, *p* < 0.001), and FSS (β = 0.675, *p* < 0.001) (Table 7). Sleep disturbances (PSQI5) also showed significant associations with all three outcomes: HADS-A (β = 1.161, *p* = 0.002), HADS-D (β = 0.833, *p* = 0.017), and FSS (β = 0.426, *p* = 0.003). Similarly, use of sleep medication (PSQI6) was a significant predictor of HADS-A (β = 0.658, *p* = 0.001), HADS-D (β = 0.668, *p* = 0.001), and FSS (β = 0.169, *p* = 0.035).

Other components such as subjective sleep quality (PSQI1) and sleep duration (PSQI3) were not significantly associated with any of the HADS-A, HADS-D, and FSS (*p* > 0.05), except for a marginal association between PSQI1 and HADS-D (β = 0.487, *p* = 0.081). Among demographic covariates, age was significantly associated only with HADS-D (β = 0.036, *p* = 0.044), while no significant associations were observed for HADS-A (β = −0.015, *p* = 0.414) or FSS (β = 0.010, *p* = 0.163). Sex emerged as a borderline-significant predictor of FSS (β = −0.388, *p* = 0.057), suggesting lower FSS scores among males, while demonstrating no predictive utility for HADS-A (β= −0.792, *p* = 0.130) or HADS-D (β = 0.287, *p* = 0.561) in fully adjusted models. EDSS was a significant predictor of HADS-D scores (β = 0.383, *p* < 0.001) and FSS (β = 0.161, *p* < 0.001), but not HADS-A (β = 0.101, *p* = 0.302). The final models explained 37.2% of the variance in HADS-A, 42.7% in HADS-D, and 32.6% in FSS (Table 7).

## 4. Discussion

The present study confirms that MS subjects experience significantly higher levels of fatigue, depressive symptoms, and poor sleep quality compared to control subjects, while anxiety levels do not differ significantly between groups. These results are consistent with previous research highlighting the high prevalence of non-motor symptoms in MS, such as depression, anxiety, fatigue, and sleep disturbances, which often co-occur and exacerbate the overall impact of the MS [3,16,17,18,19,21,27,50].

The study demonstrated robust intercorrelations between FSS, HADS-D, and PSQI global scores, and a positive association of FSS, HADS-D, and PSQI with EDSS severity. However, the correlations between EDSS and FSS, HADS-D, and PSQI were generally weak, which is in line with prior findings [19,27,28,29], suggesting that the EDSS, while widely used, inadequately captures the full spectrum of non-motor MS symptomatology [2,6,11,16,25,26,27,28,29,30].

Sleep quality and depression showed progressive worsening with higher EDSS severity scores, while fatigue reached its highest levels in MS subjects with moderate EDSS severity (EDSS 4.5–6.5). Anxiety levels remained consistent across all severity strata (EDSS ≤ 4; EDSS 4.5–6.5; EDSS ≥ 7), a finding that aligns with previous findings [19,51,52] and underscores the heterogeneity of anxiety symptoms in MS, as well as their potentially distinct etiological mechanisms compared to depression and fatigue [50].

The novel finding of the present study relates to sleep quality (PSQI global), found to be a robust predictor of fatigue (FSS), anxiety (HADS-A), and depression (HADS-D), even after controlling for age, sex, and disability status (EDSS). Among PSQI components, daytime dysfunction, sleep disturbances, and use of sleep medication were the most robust and consistent predictors of fatigue (FSS), depression (HADS-D), and anxiety (HADS-A) in our MS sample. These results additionally contribute to the understanding of the complex association of sleep quality with depression, anxiety, and fatigue [18,19,21,27,29]. Compared to our present study findings, previous studies reported findings on the following: (a) correlations between poor sleep, anxiety, and fatigue [19]; (b) psychometric properties of PSQI on MS samples [39]; and (c) sleep disturbances forming a unique impact on quality of life [18]. In contrast to our findings, Ozdogar et al. [19] used the version of PSQI previously not validated on the MS sample and reported on 52 MS subgroups that anxiety was significantly higher in the poor sleep quality group, with no significant differences observed for fatigue or depression. In the present study, however, we used the PSQI version previously validated on MS sample [39], and in contrast to Ozdogar et al. [19], both fatigue and depression were found to be significantly elevated among MS subjects with poorer sleep quality. Additionally, our study findings detected specific PSQI components strongly predicting depression, anxiety, and fatigue in MS, independent of EDSS score. Furthermore, Laslett et al. [18] investigated sleep quality in MS using the global PSQI score as well as applying a previously non-validated PSQI scale on the MS sample [32], and demonstrating that poorer sleep independently predicts reduced quality of life, even after accounting for symptoms such as depression and fatigue. In contrast, the present study investigated sleep quality by accounting for all PSQI components in predicting the severity of non-motor symptoms (fatigue, depression, and anxiety), while controlling disability status (EDSS). The current findings suggest that daytime dysfunction was the best predictor of fatigue, while sleep disturbances were more closely linked to anxiety. Additionally, certain aspects of sleep quality, such as the use of sleep medication, were significantly associated with the severity of depressive symptoms in MS.

Therefore, our findings further highlight that sleep problems (daytime dysfunction, sleep disturbances, and use of sleep medication) not only co-occur with depression, anxiety, and fatigue but also significantly predict their severity in MS. This underscores the clinical importance of granular, component-level sleep assessment and integrated psychological interventions to effectively address the complex interplay between sleep and non-motor symptoms in MS.

The limitations of this study should be acknowledged. The cross-sectional design precludes any inference of causality between disability status, depression, anxiety, fatigue, and sleep quality. To clarify the directionality, temporal dynamics, and complex interplay of MS disability and co-occurring non-motor symptoms, future longitudinal studies might employ objective sleep measures [19,29] and cognitive evaluation, in addition to standardized EDSS disability scoring, and validated instruments for assessing depression, anxiety, sleep quality, and fatigue in MS subjects.

Overall, the present study provide findings on: (a) moderate to strong intercorrelation between FSS, HADS-D, HADS-A and PSQI, (b) positive association of FSS, HADS-D, and PSQI with EDSS severity, (c) a steady increase in PSQI global and HADS-D scores with higher EDSS severity, (d) a pronounced elevation of FSS scores at moderate EDSS severity (EDSS 4.5–6.5), (e) generally weak correlation between EDSS and FSS, HADS-D, HADS-A and PSQI, (f) EDSS as significant predictor of HADS-D and FSS scores, (g) global PSQI score as independent predictor of FSS and HADS-D, and HADS-A, and (h) daytime dysfunction, sleep disturbances, and sleep medication use as significantly predictors of FSS, HADS-D, and HADS-A.

## 5. Conclusions

The present study demonstrates an interrelation among PSQI, FSS, and HADS-D scores, with a progressive increase in these scores observed at higher EDSS severity levels, confirming a significant association between disability status (EDSS) and non-motor symptoms (fatigue, depression, and sleep quality) in the MS sample. Daytime dysfunction, sleep disturbances, and sleep medication use were identified as consistent and robust predictors of depression, anxiety, and fatigue, independent of demographic factors and EDSS. In the end, the present study findings clarify the complex interplay between MS disease disability and non-motor symptomatology, highlighting the role of sleep quality in exacerbating depression, anxiety, and fatigue in MS.

## Figures and Tables

**Table 1:** Baseline characteristics of MS and control subjects.

	MS (*n* = 469)	Control (*n* = 369)
Age in years, mean (SD)	42.7 (10.8)	42.3 (11.7)
Age, range	18–78	18–75
Sex, *n* (%)		
Women	398 (84.8)	304 (82.4)
Men	71 (15.2)	65 (17.6)
Right-hand dominance, *n* (%)	438 (93.4)	344 (93.2)
Education, *n* (%)		
Primary school	8 (1.7)	0
Secondary school	252 (53.7)	85 (23.3)
Professional study	31 (6.6)	24 (6.3)
Undergraduate study	32 (6.9)	34 (9.2)
Graduate study	131 (27.9)	189 (51.2)
Postgraduate study	15 (3.2)	37 (10.0)
Comorbidity, *n* (%)	136 (29.0)	95 (25.7)
FSS, mean (SD)	5.01 (1.68)	4.02 (1.37)
HADS-A, mean (SD)	8.18 (4.49)	8.19 (2.97)
HADS-D, mean (SD)	7.02 (4.31)	5.48 (3.51)
PSQI global, mean (SD)	7.75 (4.04)	5.97 (3.14)
MS type, *n* (%)		-
RRMS	353 (75.3)	
PPMS	60 (12.8)	
SPMS	19 (7.8)	
CIS	2 (0.4)	
EDSS, median (Q1–Q3)	2 (1–4)	-
Duration of MS, mean (SD)	8.39 (7.74)	-
Immunomodulatory drug, *n* (%)	342 (72.9)	-

Values are presented as mean (SD) and median (Q1–Q3) for continuous variables and absolute frequency (relative frequency) for categorical variables. CIS, Clinically Isolated Syndrome; EDSS, Expanded Disability Status Scale; FSS, Fatigue Severity Scale; HADS-A, Hospital Anxiety and Depression Scale (anxiety subscale); HADS-D, Hospital Anxiety and Depression Scale (depression subscale); Q1, first quartile; Q3, third quartile; PPMS, primary progressive multiple sclerosis; PSQI, Pittsburgh Sleep Quality Index; RRMS, relapsing-remitting multiple sclerosis; SD, standard deviation; SPM, secondary progressive multiple sclerosis.

**Table 2:** Mean group comparison between MS (**n** = 469) and control (*n* = 369) subjects.

	MS Mean (SD)	Control Mean (SD)	*p*-Value
FSS	5.01 (1.68)	4.02 (1.37)	<2.2 × 10^−1^**^6^**
HADS-A	8.18 (4.49)	8.19 (2.97)	0.961
HADS-D	7.02 (4.31)	5.48 (3.51)	1.83 × 10^−8^
PSQI global	7.75 (4.04)	5.97 (3.14)	1.717 × 10^−12^

Values are presented as mean (SD). HADS-A, Hospital Anxiety and Depression Scale (anxiety subscale); HADS-D, Hospital Anxiety and Depression Scale (depression subscale); PSQI, Pittsburgh Sleep Quality Index; FSS, Fatigue Severity Scale. *p*-values represent results of independent sample *t*-tests.

**Table 3:** Pearson correlations between EDSS, FSS, HADS-A, HADS-D, and PSQI global scores in MS.

	HADS-A	HADS-D	PSQI Global	EDSS
FSS	0.517 (<0.001)	0.577 (<0.001)	0.399 (<0.001)	0.266 (<0.001)
HADS-A	-	0.665 (<0.001)	0.474 (<0.001)	0.095 (0.056)
HADS-D	-	-	0.466 (<0.001)	0.294 (<0.001)
PSQI global	-	-	-	0.153 (0.004)

Values represent Pearson correlation coefficients with corresponding *p*-values shown in parentheses. HADS-A, Hospital Anxiety and Depression Scale (anxiety subscale); HADS-D, Hospital Anxiety and Depression Scale (depression subscale); PSQI, Pittsburgh Sleep Quality Index; FSS, Fatigue Severity Scale, EDSS, Expanded Disability Status Scale.

**Table 4:** Hierarchical linear regression models predicting HADS-A from PSQI global and covariates.

Predictor Variables	Dependent Variable: HADS-A		
	(1)	(2)	(3)
PSQI global	0.528 (<0.001)	0.519 (<0.001)	0.527 (<0.001)
Age		−0.022 (0.227)	−0.026 (0.193)
Sex (Male)		−1.072 (0.05)	−1.033 (0.069)
EDSS			0.122 (0.249)
Observations	461	441	386
Adjusted R^2^	0.223	0.220	0.240
F-statistic	132.812 (df = 1; 459)	42.362 (df = 3; 437)	31.467 (df = 4; 381)

Values represent regression coefficients with corresponding *p*-values in parentheses. EDSS, Expanded Disability Status Scale; HADS-A, Hospital Anxiety and Depression Scale (anxiety subscale); PSQI, Pittsburgh Sleep Quality Index. Model 1 includes PSQI only; Model 2 adds age and sex; Model 3 adds EDSS. *p*-values < 0.05 are considered statistically significant and are marker in bold text.

**Table 5:** Hierarchical linear regression models predicting HADS-D from PSQI global and covariates.

Predictor Variables	Dependent Variable: HADS-D		
	(1)	(2)	(3)
PSQI global	0.499 (<0.001)	0.489 (<0.001)	0.480 (<0.001)
Age		0.051 (0.003)	0.028 (0.147)
Sex (Male)		0.505 (0.326)	0.148 (0.788)
EDSS			0.435 (<0.001)
Observations	461	441	386
Adjusted R^2^	0.215	0.221	0.263
F-statistic	127.094 (df = 1; 459)	42.593 (df = 3; 437)	35.434 (df = 4; 381)

Values represent regression coefficients with corresponding *p*-values in parentheses. EDSS, Expanded Disability Status Scale; HADS-D, Hospital Anxiety and Depression Scale (depression subscale); PSQI, Pittsburgh Sleep Quality Index. Model 1 includes PSQI only; Model 2 adds age and sex; Model 3 adds EDSS. *p*-values < 0.05 are considered statistically significant and are marked in bold text.

**Table 6:** Hierarchical linear regression models predicting FSS from PSQI global and covariates.

Predictor Variables	Dependent Variable: FSS		
	(1)	(2)	(3)
PSQI global	0.166 (<0.001)	0.153 (<0.001)	0.151 (<0.001)
Age		0.017 (0.018)	0.009 (0.227)
Sex (Male)		−0.266 (0.195)	− 0.484 (0.024)
EDSS			0.170 (<0.001)
Observations	464	445	390
Adjusted R^2^	0.157	0.150	0.212
F-statistic	87.288 (df = 1; 462)	27.030 (df = 3; 441)	27.114 (df = 4; 385)

Values represent regression coefficients with corresponding *p*-values in parentheses. EDSS, Expanded Disability Status Scale; PSQI, Pittsburgh Sleep Quality Index; FSS, Fatigue Severity Scale. Model 1 includes PSQI only; Model 2 adds age and sex; Model 3 adds EDSS. *p*-values < 0.05 are considered statistically significant and are marked in bold text.

**Table 7:** Multiple regression models analyzing PSQI subscales as predictors of HADS-A, HADS-D, and FSS and covariates.

Predictor Variables	Dependent Variable		
	HADS-A	HADS-D	FSS
PSQI1 (Subjective quality)	0.545 (0.066)	0.487 (0.081)	0.129 (0.267)
PSQI2 (Sleep latency)	0.064 (0.775)	0.026 (0.903)	0.003 (0.969)
PSQI3 (Sleep duration)	0.106 (0.715)	−0.353 (0.198)	−0.140 (0.218)
PSQI4 (Sleep efficiency)	0.044 (0.845)	0.256 (0.228)	0.067 (0.445)
PSQI5 (Sleep disturbances)	1.161 (0.002)	0.833 (0.017)	0.426 (0.003)
PSQI6 (Use of sleep medications)	0.658 (0.001)	0.668 (0.001)	0.169 (0.035)
PSQI7 (Daytime dysfunction)	1.990 (<0.001)	2.220 (<0.001)	0.675 (<0.001)
Age	−0.015 (0.414)	0.036 (0.044)	0.010 (0.163)
Sex (Male)	−0.792 (0.130)	0.287 (0.561)	−0.388 (0.057)
EDSS	0.101 (0.302)	0.383 (<0.001)	0.161 (<0.001)
Observations	373	373	377
Adjusted R^2^	0.372	0.427	0.326
F-statistic	23.081 (df = 10; 362)	28.726 (df = 10; 362)	19.183 (df = 10; 366)

Values represent regression coefficients with corresponding *p*-values in parentheses. All models are adjusted for age, sex, and EDSS. *p*-values < 0.05 are considered statistically significant and are marked in bold text. EDSS, Expanded Disability Status Scale; FSS, Fatigue Severity Scale; HADS-A, Hospital Anxiety and Depression Scale (anxiety subscale); HADS-D, Hospital Anxiety and Depression Scale (depression subscale); PSQI, Pittsburgh Sleep Quality Index.

## Data Availability

The data presented in this study are available on request from the corresponding author. The data are not publicly available due to privacy restrictions.

## Abbreviations

The following abbreviations are used in this manuscript:

AMSSC	Association of Multiple Sclerosis Societies of Croatia
CIS	Clinically Isolated Syndrome
CNS	Central Nervous System
EDSS	Expanded Disability Status Scale
FS	Functional Score
FSS	Fatigue Severity Scale
HADS	Hospital Anxiety and Depression Scale
HADS-A	Hospital Anxiety and Depression Scale (anxiety subscale)
HADS-D	Hospital Anxiety and Depression Scale (depression subscale)
MS	Multiple Sclerosis
PSQI	Pittsburgh Sleep Quality Index

## References

[1] Bjørklund G., Wallace D.R., Hangan T., Butnariu M., Gurgas L., Peana M. (2025). Cerebral Iron Accumulation in Multiple Sclerosis: Pathophysiology and Therapeutic Implications. Autoimmun. Rev..

[2] Geurts J.J., Calabrese M., Fisher E., Rudick R.A. (2012). Measurement and Clinical Effect of Grey Matter Pathology in Multiple Sclerosis. Lancet Neurol..

[3] Lorenzut S., Negro I.D., Pauletto G., Verriello L., Spadea L., Salati C., Musa M., Gagliano C., Zeppieri M. (2025). Exploring the Pathophysiology, Diagnosis, and Treatment Options of Multiple Sclerosis. J. Integr. Neurosci..

[4] Magliozzi R., Howell O.W., Calabrese M., Reynolds R. (2023). Meningeal Inflammation as a Driver of Cortical Grey Matter Pathology and Clinical Progression in Multiple Sclerosis. Nat. Rev. Neurol..

[5] Peruzzotti-Jametti L., Willis C.M., Krzak G., Hamel R., Pirvan L., Ionescu R.-B., Reisz J.A., Prag H.A., Garcia-Segura M.E., Wu V. (2024). Mitochondrial Complex I Activity in Microglia Sustains Neuroinflammation. Nature.

[6] Woo M.S., Mayer C., Binkle-Ladisch L., Sonner J.K., Rosenkranz S.C., Shaposhnykov A., Rothammer N., Tsvilovskyy V., Lorenz S.M., Raich L. (2024). STING Orchestrates the Neuronal Inflammatory Stress Response in Multiple Sclerosis. Cell.

[7] Glaser A., Stahmann A., Meissner T., Flachenecker P., Horáková D., Zaratin P., Brichetto G., Pugliatti M., Rienhoff O., Vukusic S. (2019). Multiple Sclerosis Registries in Europe—An Updated Mapping Survey. Mult. Scler. Relat. Disord..

[8] Declemy A., Haddad R., Chesnel C., Charlanes A., Le Breton F., Sheikh Ismael S., Amarenco G. (2021). Prevalence of Comorbidities in Multiple Sclerosis Patients with Neurogenic Bladder. Prog. Urol..

[9] Rodrigues P., Da Silva B., Trevisan G. (2023). A Systematic Review and Meta-Analysis of Neuropathic Pain in Multiple Sclerosis: Prevalence, Clinical Types, Sex Dimorphism, and Increased Depression and Anxiety Symptoms. Neurosci. Biobehav. Rev..

[10] Benedict R.H., Amato M.P., Boringa J., Brochet B., Foley F., Fredrikson S., Hamalainen P., Hartung H., Krupp L., Penner I. (2012). Brief International Cognitive Assessment for MS (BICAMS): International Standards for Validation. BMC Neurol..

[11] Benedict R.H.B., Amato M.P., DeLuca J., Geurts J.J.G. (2020). Cognitive Impairment in Multiple Sclerosis: Clinical Management, MRI, and Therapeutic Avenues. Lancet Neurol..

[12] Cordani C., Meani A., Esposito F., Valsasina P., Colombo B., Pagani E., Preziosa P., Comi G., Filippi M., Rocca M.A. (2020). Imaging Correlates of Hand Motor Performance in Multiple Sclerosis: A Multiparametric Structural and Functional MRI Study. Mult. Scler. J..

[13] Manjaly Z.-M., Harrison N.A., Critchley H.D., Do C.T., Stefanics G., Wenderoth N., Lutterotti A., Müller A., Stephan K.E. (2019). Pathophysiological and Cognitive Mechanisms of Fatigue in Multiple Sclerosis. J. Neurol. Neurosurg. Psychiatry.

[14] Rocca M.A., Preziosa P., Barkhof F., Brownlee W., Calabrese M., De Stefano N., Granziera C., Ropele S., Toosy A.T., Vidal-Jordana À. (2024). Current and Future Role of MRI in the Diagnosis and Prognosis of Multiple Sclerosis. Lancet Reg. Health—Eur..

[15] Jellinger K.A. (2024). Depression and Anxiety in Multiple Sclerosis. Review of a Fatal Combination. J. Neural Transm..

[16] Karimi S., Andayeshgar B., Khatony A. (2020). Prevalence of Anxiety, Depression, and Stress in Patients with Multiple Sclerosis in Kermanshah-Iran: A Cross-Sectional Study. BMC Psychiatry.

[17] Oliva Ramirez A., Keenan A., Kalau O., Worthington E., Cohen L., Singh S. (2021). Prevalence and Burden of Multiple Sclerosis-Related Fatigue: A Systematic Literature Review. BMC Neurol..

[18] Laslett L.L., Honan C., Turner J.A., Dagnew B., Campbell J.A., Gill T.K., Appleton S., Blizzard L., Taylor B.V., Van Der Mei I. (2022). Poor Sleep and Multiple Sclerosis: Associations with Symptoms of Multiple Sclerosis and Quality of Life. J. Neurol. Neurosurg. Psychiatry.

[19] Ozdogar A.T., Aldemir E., Yesiloglu P., Cilingir V. (2025). Exploring the Relationship Between Sleep Quality and Fatigue, Quality of Life, Daytime Sleepiness, and Anxiety-Depression Levels in Patients with Multiple Sclerosis. J. Mult. Scler. Res..

[20] Zhang G.X., Zhang W.T., Gao S.S., Zhao R.Z., Yu W.J., Izquierdo G. (2024). Sleep Disorders in Patients with Multiple Sclerosis in Spain. Neurol. Engl. Ed..

[21] Zekibakhsh Mohammadi N., Kianimoghadam A.S., Mikaeili N., Asgharian S.S., Jafari M., Masjedi-Arani A. (2024). Sleep Disorders and Fatigue among Patients with MS: The Role of Depression, Stress, and Anxiety. Neurol. Res. Int..

[22] Curatoli C., Marcassoli A., Barbadoro F., Fornari A., Leonardi M., Raggi A., Schiavolin S., Terragni R., Antozzi C., Brambilla L. (2025). Anxiety, Depression, and Expanded Disability Status Scale Independently Predict the Perception of Disability in Persons with Multiple Sclerosis: A Cross-Sectional Study. Behav. Neurol..

[23] D’Souza M., Heikkilä A., Lorscheider J., Haller V., Kravalis K., Gysin S., Fuertes N.A.C., Fricker E., Lam E., Higgins P. (2020). Electronic Neurostatus-EDSS Increases the Quality of Expanded Disability Status Scale Assessments: Experience from Two Phase 3 Clinical Trials. Mult. Scler. J..

[24] Kurtzke J.F. (1983). Rating Neurologic Impairment in Multiple Sclerosis: An Expanded Disability Status Scale (EDSS). Neurology.

[25] Asadollahzadeh E., Ebadi Z., Owji M., Rezaeimanesh N., Sahraian M.A., Moghadasi A.N. (2024). Exploring the Relationship between Disability Status, Depression, and Quality of Life in Individuals with Multiple Sclerosis. Mult. Scler. Relat. Disord..

[26] Martínez-Ginés M.L., Esquivel A., Hernández Y.H., Alvarez-Sala L.A., Benito-León J. (2024). Investigating the Relationship between Multiple Sclerosis Disability and Driving Performance: A Comparative Study of the Multiple Sclerosis Functional Composite and Expanded Disability Status Scale. Clin. Neurol. Neurosurg..

[27] Aparicio Castro E., Candeliere Merlicco A., María Santa C., Villaverde González R. (2024). Utilidad de la escala de depresión de Beck para el diagnóstico de los trastornos depresivos en la esclerosis múltiple. Rev. Neurol..

[28] Ezzeldin M.Y., Mahmoud D.M., Safwat S.M., Soliman R.K., Desoky T., Khedr E.M. (2023). EDSS and Infratentorial White Matter Lesion Volume Are Considered Predictors of Fatigue Severity in RRMS. Sci. Rep..

[29] Riccitelli G.C., Disanto G., Sacco R., Sparasci D., Sacco L., Castelnovo A., Miano S., Manconi M., Gobbi C., Zecca C. (2021). Contribution of Sleep Disturbances to Fatigue in Multiple Sclerosis: A Prospective Study Using Clinical and Polysomnographic Parameters. Eur. J. Neurol..

[30] Alswat A.M., Altirkistani B.A., Alserihi A.R., Baeshen O.K., Alrushid E.S., Alkhudair J., Aldbas A.A., Wadaan O.M., Alsaleh A., Al Malik Y.M. (2023). The Prevalence of Major Depression and Generalized Anxiety Disorder in Patients with Multiple Sclerosis in Saudi Arabia: A Cross-Sectional Multicentered Study. Front. Psychiatry.

[31] Kołtuniuk A., Kazimierska-Zając M., Pogłódek D., Chojdak-Łukasiewicz J. (2022). Sleep Disturbances, Degree of Disability and the Quality of Life in Multiple Sclerosis Patients. Int. J. Environ. Res. Public Health.

[32] Buysse D.J., Reynolds C.F., Monk T.H., Berman S.R., Kupfer D.J. (1989). The Pittsburgh Sleep Quality Index: A New Instrument for Psychiatric Practice and Research. Psychiatry Res..

[33] Zigmond A.S., Snaith R.P. (1983). The Hospital Anxiety and Depression Scale. Acta Psychiatr. Scand..

[34] Krupp L.B. (1989). The Fatigue Severity Scale: Application to Patients with Multiple Sclerosis and Systemic Lupus Erythematosus. Arch. Neurol..

[35] McDonald W.I., Compston A., Edan G., Goodkin D., Hartung H., Lublin F.D., McFarland H.F., Paty D.W., Polman C.H., Reingold S.C. (2001). Recommended Diagnostic Criteria for Multiple Sclerosis: Guidelines from the International Panel on the Diagnosis of Multiple Sclerosis. Ann. Neurol..

[36] Thompson A.J., Banwell B.L., Barkhof F., Carroll W.M., Coetzee T., Comi G., Correale J., Fazekas F., Filippi M., Freedman M.S. (2018). Diagnosis of Multiple Sclerosis: 2017 Revisions of the McDonald Criteria. Lancet Neurol..

[37] Ayache S.S., Chalah M.A. (2017). Fatigue in Multiple Sclerosis—Insights into Evaluation and Management. Neurophysiol. Clin. Neurophysiol..

[38] Feldpausch J., Plummer P., Abou-Rass Z., Fritz N. (2024). Characterizing Fatigue by Multiple Sclerosis Subtype and Determining Validity of a Fatigue Scale Specific to Persons with Progressive Multiple Sclerosis. Int. J. MS Care.

[39] Jerković A., Mikac U., Matijaca M., Košta V., Ćurković Katić A., Dolić K., Vujović I., Šoda J., Đogaš Z., Pavelin S. (2022). Psychometric Properties of the Pittsburgh Sleep Quality Index (PSQI) in Patients with Multiple Sclerosis: Factor Structure, Reliability, Correlates, and Discrimination. J. Clin. Med..

[40] Armutlu K., Cetisli Korkmaz N., Keser I., Sumbuloglu V., Irem Akbiyik D., Guney Z., Karabudak R. (2007). The Validity and Reliability of the Fatigue Severity Scale in Turkish Multiple Sclerosis Patients. Int. J. Rehabil. Res..

[41] Lerdal A., Johansson S., Kottorp A., Von Koch L. (2010). Psychometric Properties of the Fatigue Severity Scale: Rasch Analyses of Responses in a Norwegian and a Swedish MS Cohort. Mult. Scler. J..

[42] Valko P.O., Bassetti C.L., Bloch K.E., Held U., Baumann C.R. (2008). Validation of the Fatigue Severity Scale in a Swiss Cohort. Sleep.

[43] Honarmand K., Feinstein A. (2009). Validation of the Hospital Anxiety and Depression Scale for Use with Multiple Sclerosis Patients. Mult. Scler. J..

[44] Marrie R.A., Zhang L., Lix L.M., Graff L.A., Walker J.R., Fisk J.D., Patten S.B., Hitchon C.A., Bolton J.M., Sareen J. (2018). The Validity and Reliability of Screening Measures for Depression and Anxiety Disorders in Multiple Sclerosis. Mult. Scler. Relat. Disord..

[45] Pais Ribeiro J.L., Martins Da Silva A., Vilhena E., Moreira I., Santos E., Mendonça D. (2018). The Hospital Anxiety and Depression Scale, in Patients with Multiple Sclerosis. Neuropsychiatr. Dis. Treat..

[46] Watson T.M., Ford E., Worthington E., Lincoln N.B. (2014). Validation of Mood Measures for People with Multiple Sclerosis. Int. J. MS Care.

[47] Jerković A., Proroković A., Matijaca M., Vuko J., Poljičanin A., Mastelić A., Ćurković Katić A., Košta V., Kustura L., Dolić K. (2021). Psychometric Properties of the HADS Measure of Anxiety and Depression Among Multiple Sclerosis Patients in Croatia. Front. Psychol..

[48] Curcio G., Tempesta D., Scarlata S., Marzano C., Moroni F., Rossini P.M., Ferrara M., De Gennaro L. (2013). Validity of the Italian Version of the Pittsburgh Sleep Quality Index (PSQI). Neurol. Sci..

[49] Team R.C. R Foundation for Statistical Computing.

[50] Dogan S., Yildiz S., Kazgan Kılıçaslan A., Sirlier Emir B., Kurt O., Sehlikoğlu S. (2024). Does Anxiety, Depression, and Sleep Levels Affect the Quality of Life in Patients Diagnosed with Multiple Sclerosis?. Eur. Rev. Med. Pharmacol. Sci..

[51] Beiske A.G., Svensson E., Sandanger I., Czujko B., Pedersen E.D., Aarseth J.H., Myhr K.M. (2008). Depression and Anxiety amongst Multiple Sclerosis Patients. Eur. J. Neurol..

[52] Dahl O.-P., Stordal E., Lydersen S., Midgard R. (2009). Anxiety and Depression in Multiple Sclerosis. A Comparative Population-Based Study in Nord-Trøndelag County, Norway. Mult. Scler. J..

